# An Ailment with Which I Will Contend: A Narrative Review of 5000 Years of Esophagogastric Cancers and Their Treatments, with Special Emphasis on Recent Advances in Immunotherapeutics

**DOI:** 10.3390/cancers16030618

**Published:** 2024-01-31

**Authors:** C. Beau Hilton, Steven Lander, Michael K. Gibson

**Affiliations:** 1 Vanderbilt Ingram Cancer Center, Vanderbilt University Medical Center, 2220 Pierce Ave, Nashville, TN 37232, USA; 2 Internal Medicine Residency Program, University of Tennessee Health Sciences Center, 920 Madison Ave, Suite 531, Memphis, TN 38163, USA; slander2@uthsc.edu

**Keywords:** checkpoint inhibitors, esophageal cancer, gastric cancer, immunotherapy, PD-L1, HER2, clinical trials, history, disparities

## Abstract

**Simple Summary:**

Cancers of the esophagus and stomach are among the most common and deadly cancers worldwide, and have been major contributors to human suffering throughout recorded history. This review traces the chronology of these cancers from 3000 BCE to the present. The first several thousand years were devoted to palliative operations, before advances in operative technique and technology led to the first curative surgery in 1913. Around the same time, in 1910, systemic therapies were introduced, and radiotherapy shortly thereafter. The later decades of the 20th century saw a groundswell of advances in surgery, endoscopic therapy, systemic therapy, and radiotherapy, and, from the 1970s, an increasing focus on combination approaches. While studies in these areas continued to advance, immunotherapies became a major new category in the late 2010s. An increasing number of immunotherapies have been developed and now encompass more indications and earlier lines of therapy. As standards of care incorporate these effective yet expensive therapies, care must be given to disparities and methods for increasing access.

**Abstract:**

Esophagogastric cancers are among the most common and deadly cancers worldwide. This review traces their chronology from 3000 BCE to the present. The first several thousand years were devoted to palliation, before advances in operative technique and technology led to the first curative surgery in 1913. Systemic therapies were introduced in 1910, and radiotherapy shortly thereafter. Operative technique improved massively over the 20th century, with operative mortality rates reducing from over 50% in 1933 to less than 5% by 1981. In addition to important roles in palliation, endoscopy became a key nonsurgical curative option for patients with limited-stage disease by the 1990s. The first nonrandomized studies on combination therapies (chemotherapy ± radiation ± surgery) were reported in the early 1980s, with survival benefit only for subsets of patients. Randomized trials over the next decades had similar overall results, with increasing nuance. Disparate conclusions led to regional variation in global practice. Starting with the first FDA approval in 2017, multiple immunotherapies now encompass more indications and earlier lines of therapy. As standards of care incorporate these effective yet expensive therapies, care must be given to disparities and methods for increasing access.

## 1. Introduction

As the seventh most common cause of cancer worldwide, and the sixth leading cause of cancer mortality, esophageal cancer is a prominent disease that affects almost every population [1]. Cancers of the stomach (GCs) are the fifth most common, and fourth in mortality. Though distinct in many ways from EC proper, in causes, biology, and sociodemographic distribution, GCs and gastroesophageal junction cancers (GEJCs) are often included with EC in clinical trials and clinical practice, and are sometimes difficult to disentangle from EC in global statistics—if considered together, worldwide, esophagogastric cancers (EGCs) are the fourth most common overall, and rank third in mortality (lung and breast cancers are, respectively, the first and second both in incidence and mortality) [1]. In this review, we focus largely on EC, but recognize the overlaps with GC and GEJC, and note distinctions as appropriate.

There are two common types of EC: squamous cell carcinoma (SCC) and adenocarcinoma (AC). SCC predominates globally and AC is more common in Western nations. In the mid-century, ESCC accounted for over 90 percent of cases worldwide. This has shifted over time to a higher incidence of AC, particularly in Western (and Westernized) countries. This is due in part to lower incidences of causative factors for ESCC: cigarette smoking, incidental inhalational exposure, nutritional deficiencies, and chronic *Helicobacter Pylori* infections, with increasing incidences of causative factors for EAC: obesity and downstream effects on gastroesophageal reflux disease and Barrett’s esophagus [2]. Screening programs and increased access to healthcare have helped with earlier diagnosis and management, but over 50% of patients still present with metastases already established [3].

Management of EC is often multimodal. Surgical and endoscopic resection and ablation are preferred for localized and locoregional disease, with varying contributions of chemotherapy and radiation, depending on specific patient, disease, and sociogeographic features (i.e., local preferences and availability of therapies). Multidrug chemotherapy regimens are common in both localized and metastatic settings, and, in recent years, immunotherapy (IO) and other targeted therapies (including hormonal therapy) have grown in prevalence and importance.

This chronological narrative review traces the history of EC, from the earliest times to the present, with a focus on changes in patient outcomes with increasing expertise and new (bio)technologies including immunotherapy. Disparities are highlighted and areas for improvement suggested.

## 2. Early History

There are hints of EC throughout the extent of recorded history (see Table 1 for a summary). The first recorded surgical intervention of the esophagus was found in an Egyptian document from approximately 3000 BCE, in which the surgeon describes “One having a wound in his throat, piercing through to his gullet. An ailment with which I will contend” [4]. The second sentence is a classification, in a system where “an ailment which I will treat” denotes a curable problem, “an ailment with which I will contend” denotes potential for cure, and “an ailment which will not be treated” denotes incurability [5]. The Egyptian surgeon proceeds to describe various procedures including suturing the wound, observing for infection, and dressing it as needed with meat, honey, cloth, etc. [4].

Records from China beginning around 0 BCE describe swallowing and belching disorders due to esophageal masses, and recognize the contribution of lifestyle factors including “heated liquors,” as well as increasing incidence due to age [4,6]. The disease was common, as were poor outcomes: “those discovered to suffer in the autumn… will not live through the next summer.” [6] It garnered sufficient cultural respect to the degree that a temple called the Houwang Miao (“Throat God Temple”) was built some time in the first millennium (and was, unfortunately, destroyed in 1927) [7].

In the West, the 2nd century Roman Greek physician Galen was the first to record “fleshy growths” of the esophagus and associated poor prognoses [4]. Ibn Zuhr (transliterated as “Avenzoar” in most Western literature), an 11–12th century Arabian physician, described EC and recommended methods for palliation, including silver esophagogastric feeding tubes and nutritive enemata [4,5,7]. Most of the descriptions from this time until the mid-1800s were focused on anatomy, the dire nature of the disease, and possible etiologies. Treatments were minimal if any and always palliative.

The most striking description from this period comes from English surgeon John Casaubon, who became afflicted with EC himself and recorded his decline in a single entry in his diary in the last week of 1690, just prior to his death in 1691. This entry was reproduced in partially commuted form in several prior publications [4,6]. The archivist at the Southampton City Archives in the United Kingdom kindly provided us with a scan of the original text, which was newly transcribed by the authors and is reproduced here in its entirety for the first time. While the text itself is ancient and therefore in the public domain, the copyright for the microfilm scan of the diary is owned by the British Library and therefore the photograph itself cannot be reproduced here.

Monday December 29. 90

At dinner I was almost choaked by swallowing a bit of a roasted Sd of mutton which as I thought stuck in the passage about the mouth of the stomach. But it suffered noething to goe downe and the stomach threw all up, though never soe small in quantitie, to all our amazements the sckilfull not knowing what 2 make of my condition. It being an unusuall afflixion wch. my melancholi suggested it an extraordinarie judgment. I could swallow about 2 spoonfulls about half way (as I thought) and then it would flush up in spite of my hart. Some small humiditie or dropps of what I dranck, rather distilld, or dropt into the stomach which afforded meate doeth noe gt. good and I am in a kind of atrophie. What warme weather may do I cant’ tell, but hope well. Alwayes after I have bine at Stoole I am for a whyle very faint or weake which I much wonder at. It is a sine of gt weakenes certainly and of insoaed decay [8].

(The remainder of the page is blank, and the diary ends here).

 

The next two notable developments were epidemiologic, rather than personal, and are both the European recognition of ideas established nearly two millennia earlier in China: Ernst Gottfried Gyser was the first European to suggest a link between heavy alcohol use and EC, which he published in his doctoral dissertation on EC in 1770 in Strasbourg, France [9], and John Howship, a British physician, was the first European to suggest, in the early 1800s, a link between age and EC [4].

## 3. Surgical Advancements

The mid-1800s to mid-1900s saw a significant growth in tools and procedures to diagnose, palliate, and, in at least one case, cure EC. Albrecht Theodor von Middeldorpf, a surgeon in Breslau, performed the first recorded EC surgery in 1857 (not counting the Egyptian document or some other nonspecific reports on, e.g., esophagotomy to palliate strictures of unknown causes) [4]. Nine years later, in 1868, Adolf Kussmaul invented the esophagoscope and successfully interrogated the esophagus (of a volunteer sword-swallower) all the way through to the stomach [7]. Shortly after this, in 1872, the first known full esophagectomy was performed by Theodor Billroth in Austria, assisted by Vincenz Czerny [4,5,6]. Czerny went on to successfully resect the cervical esophagus in 1877, and the patient achieved a postoperative survival of 15 months. Czerny was also one of the first physicians to recognize the dismal long-term survival for many cancers even with successful resection and pioneered experiments in multimodal therapy including vaccine therapy (which can be thought of as an early attempt at IO), radiotherapy (RT), and chemotherapy. For example, arsphenamine, the first agent specifically called a “chemotherapy,” was initially developed in 1909 to treat syphilis and remained the standard of care for this disease until the advent of penicillin. It was adopted as a cancer therapy by Czerny’s group in 1910 [10,11]. In 1913, the first reported cure of EC was achieved: Torek successfully resected a midesophageal EC via left thoracotomy, with a postoperative survival of 12 years [12,13]. The patient was fed via cervical esophagostomy connected extracorporeally to a gastrostomy by a rubber tube. After initial recovery, she was offered a more cosmetic reconstruction, but refused, and died of pneumonia at the age of 80 [14]. The standard of care in the 1920s and 1930s in most Western countries was palliative bypass, e.g., via gastrostomy [4].

Beginning in the 1930s, data on EC advanced from case reports to formal series. Oshawa, a Japanese surgeon, was perhaps the first to develop extensive experience with EC resections, reporting on 18 thoracic EC resections in 1933, with a 56% mortality rate (notably, this publication was not available in the West until after World War II [5]) [15]. Ochsner and DeBakey scoured world literature in 1940, found 191 EC resections reported, and noted an overall 71.7% mortality rate [16]. By 1947, Sweet was able to report personally on 213 EC resections, with a 17% mortality, a major improvement, but a 5-year survival of 8% [4]. The Japanese surgical establishment continued, as it does to this day, to have the most extensive experience and best comparative mortality rates—Nakayama reported on 953 resections, with an improvement in mortality to 5.8%, in 1959 [17]. In the West in 1963, Logan reported on a similar number of resections, 853, but with a mortality rate of 29%, and 5-year survival of 6% (much smaller reports were generated in 1960, 1961, and 1962 by several Western surgeons, with similar results) [18,19]. In 1981, Akiyama reported on 210 resections, with a 59.3% operability rate, a 1.4% mortality, and a 34.6% 5-year survival [20]. By the 1980s, mortality rates in the West improved to the mid-teens (Müller reported a 56% operability rate, with a 13% mortality [21]), and further to the single-digits (Hurt reported a 5–11% operative mortality in 1991 [15]), but long-term mortality improved little if at all: a 5-year survival of 14–22% [15].

While the remainder of this review focuses on minimally invasive, systemic, and combination therapies, it is worth noting that surgical technique has continued to advance to the modern age. For example, a 2019 trial of 112 patients found that newer surgical approaches with robot-assisted minimally invasive thoracolaparoscopic esophagectomy (RAMIE) proffered faster recovery, fewer complications, and improved quality of life compared to open transthoracic esophagectomy [22].

## 4. Advances in Endoscopic Therapies

In addition to traditional surgical approaches, endoscopy has grown to play a major role in the modern management of EGCs.

Laying the groundwork for modern endoscopic thermal therapies, neodymium:yttrium aluminum garnet (Nd:YAG) lasers were first used preclinically in an endoscope in 1973 [23], and then clinically in 1975 for ablation of bleeding colonic hemangiomata and gastric ulcers [24] (after a 1987 report in the New England Journal of Medicine showed a lack of improvement in overall outcomes, combined with a high cost of obtaining the laser, this technique fell out of favor for gastric bleeds [25]). The first endoscopic laser therapies specifically for EC were Nd:YAG approaches described by Fleischer and Kressler in 1983, used to palliate advanced cases. With a mean of 5.3 treatments, 90% of patients achieved luminal patency, and 70% of patients were able to regain the ability to sustain themselves orally [26] (since that time, esophageal stents, argon plasma laser therapy, and photodynamic therapy have become preferred endoscopic palliation methods for obstruction, in addition to nonendoscopic methods such as RT, though Nd:YAG is still occasionally used [27]). Building on the success of palliative endoscopic thermal therapy with Nd:YAG, photodynamic therapy (PDT), which has been around in one form or another since the early 1900s, with refinement and new areas of application occurring in fits and starts every few decades, was officially approved in Japan as a curative-intent therapy for superficial EC and GCs (and early-stage lung and cervical cancers) in 1994 [28]. Radiofrequency ablation (RFA) was introduced by a multidisciplinary team of doctors of veterinary and human medicine in 2004, with a three-phase run-in study with 51 swine followed by a fourth phase with RFA applied to three human patients immediately prior to traditional resection [29]. It is a preferred method for treating Barrett’s Esophagus [30,31], alongside liquid nitrogen spray cryotherapy and the EMR/ESD techniques described below, and so plays an important role in reducing EC incidence and improving overall outcomes. As can be seen by the progression of thermal therapies just described, and as is so often the case in medicine, techniques pioneered on the sickest, for whom no options other than experimentation remained, eventually led to widespread tools that benefit many.

Endoscopic mucosal resection (EMR) was also initially developed decades before its widespread use, first in the 1950s, with a sharp ramp upward in interest in the 1980s on its way to prevalence in the 1990s. EMR is a collection of methods, often including an injection of fluid or gel within the submucosal layer to dissect a plane and lift lesions for resection, that has proven highly effective for early-stage cancers depending on size, depth, location, and endoscopist experience [32,33]. Inoue introduced the endoscopic mucosal resection with a cap (EMR-C) technique in 1990, a major advance that balanced the greater effectiveness of endoscopic submucosal dissection (ESD, which had been introduced in the late 1980s [34]) with the increased safety of EMR [35]. ESD had a resurgence in the late 1990s in Japan, largely owing to technological advances and the desire to push the boundary on which lesions could be cured nonsurgically [36,37,38,39]. ESD later gained popularity worldwide [40], and refinements to EMR and ESD continue to the current day [41].

## 5. Combined Modality Advances

Recognizing poor long-term outcomes even with increasing worldwide surgical expertise throughout the mid-century, and with the further development of chemotherapeutics and improvements in RT, groups in the 1970s began to systematically study pre- and perioperative strategies for improving surgical outcomes and long-term survival. Neoadjuvant RT, which had been used for EC since at least the 1960s but only reported on retrospectively (e.g., Nakayama’s group used 20–30 Gy preoperatively and reported a 5-year survival [5y-OS] of 37.5%, compared to 19.1% with surgery alone and 7.7% with radiation alone) [18], was formally trialed first in the mid-1970s, and reported in 1981 by Launois et al.: the results were negative [42]. The first reported neoadjuvant chemoradiotherapy (CRT) trial for EC was performed at Wayne State University in Detroit, Michigan, and published in 1981 [43]. In this nonrandomized, single-arm study of 86 patients with ESCC, 30 Gy of RT and 5-fluorouracil (5-FU) plus either mitomycin-C or cisplatin were given preoperatively. In total, 92% of patients regained the ability to eat and drink within 2 weeks of CRT start, and a pathologic complete response (pCR) of 31% was achieved. The CRT-related mortality was 11%, and the operative mortality was 10%. This study was published too early to have full survival data available (a 1-year overall survival [1y-OS] of 68%, compared to the authors’ internal historical data showing 24% 1y-OS for esophagectomy alone), but follow-up publication from this group showed no overall survival benefit [44].

In 1988 Roth et al. published the first major study of preoperative chemotherapy for EC. In total, 37 of the 39 patients in the study had “epidermoid carcinoma,” an older term for ESCC, and the other 2 had undifferentiated carcinoma. They again found no benefit when the cohort was considered as a whole (*p* value 0.34), but did note that those who had response to the drugs had a median overall survival of 20 months, compared to 6.2 months for nonresponders (and 8 months for those treated with surgery alone) [45]. Similar findings, of improved outcomes for a subset and worse or unchanged outcomes for the majority, were replicated in many studies over the next decades, e.g., Schlag in 1992 [46], Maipang in 1994 [47], Law in 1997 [48], Kelsen (RTOG 8911, US Intergroup 113) in 1998/2007 [49,50], Baba in 2000 [51], and Ancona in 2001 [52]. In 2002, the Lancet published the first whole-cohort positive results for perioperative chemotherapy for EC (randomized controlled trial, administered in Britain by the Medical Research Council [MRC]) [53], and Boonstra et al. at Erasmus University Medical Center in the Netherlands published the second positive trial in 2011, with a median OS of 16 vs. 12 months and a 5-year survival of 26% vs. 17% for cisplatin and etoposide vs. surgery alone [54]. Overall, the results are heterogenous and conflicting, leading to geographically divergent treatment patterns and continued study [55,56,57].

While the above studies are mostly focused on EC proper, practicing clinicians often include studies on GCs and GEJCs in their considerations when evaluating options for patients with any of these malignancies. Two major studies that included all three: GC, GEJC, and lower EC, established perioperative chemotherapy as the standard of care, particularly in European countries. The MAGIC RCT, conducted in the United Kingdom and published in 2006, demonstrated a 5-year OS of 36% in the treatment arm (epirubicin, cisplatin, and fluorouracil, three cycles before and after surgery) vs. 23% for surgery alone, with a cohort of 503 patients with AC [58]. The Actions Concertées dans les Cancer Colorectaux et Digestifs (ACCORD) trial, a French study published in 2011, used cisplatin and fluorouracil and had nearly identical results, with an important distinction of having a predominance of GEJC and lower EC in their cohort (75%) [59]. More recently, the German FLOT-4 RCT, published in 2019, compared a modified MAGIC protocol to four pre- and postoperative cycles of fluorouracil plus leucovorin, oxaliplatin, and docetaxel, and demonstrated a median survival of 35 months for MAGIC vs. 50 months for FLOT-4 [60].

The trial with the most impact on the treatment of EC proper, particularly in the United States, remains 2012’s “ChemoRadiotherapy for Oesophageal cancer followed by Surgery Study” (CROSS) [61]. This study used preoperative carboplatin and paclitaxel with 40 Gy of radiation. The median OS was 81.6 months in the chemoradiotherapy plus surgery group vs. 21.1 months for surgery alone [62]. At 120 months of follow-up, the intervention group had a 38% survival compared with 25% in the control group. When further stratified by pathologic subtype, those with SCC (23%) who received the intervention had a 46% survival at 120 months, while those with AC (75%) had a 36% survival (the control groups had far less pathology-specific variation in survival: 26% for AC and 23% for SCC). In total, 22% of patients had GEJ disease—separate results are not available to compare GEJ and more proximal EC. In both arms and for both subtypes, no relapses occurred beyond 6 years [63]. The precise reasons for the massive relative success of the CROSS trial remain an active area of research, but speculations include a high portion of SCC (23%), better tolerability of the carboplatin/paclitaxel backbone compared to, e.g., cisplatin/5-fluorouracil backbones used in most other trials, specific susceptibility to taxanes, improvements in supportive care, etc.

## 6. Immunotherapies and Targeted Therapies

Despite improved prevention, screening, and local treatment of early-stage disease, and improved management of locally advanced disease with multimodal therapies, EC remains a deadly disease with a high inherent morbidity and difficult treatment paradigms. As such, there is an urgent need to develop new techniques and tools to optimize treatment and limit adverse effects. With the goal of improved adverse effect profiles and benefit toward morbidity and mortality end points, IO has significant promise.

Other articles in this Special Issue focus on the role of IO in the current treatment paradigm [64], so we will restrict the current discussion to the timeline of approvals and apropos changes in outcomes. The first FDA approval for IO for EGC (G and GEJ AC) was granted in 2017 for pembrolizumab based on the KEYNOTE-059 trial, initially for third-line usage only [65]. As IO moved to earlier lines of therapy, this approval was withdrawn. The next notable approval was two years later in 2019, also for pembrolizumab, but advanced to the second line. This trial, KEYNOTE-181, demonstrated an improvement in median overall survival for patients with ESCC harboring a CPS ≥ 10 to 8.2 months with pembrolizumab monotherapy compared to 7.1 months with chemotherapy [66]. Less than a year later, in mid-2020, nivolumab monotherapy gained approval for the second-line treatment of EGC agnostic of CPS, via the ATTRACTION-3 study, which showed a median survival (for unselected ESCC) of 10.9 months for IO vs. 8.4 for chemotherapy [67].

First-line approval for IO for EGC was first granted on 22 March 2021, based on KEYNOTE-590, which studied chemotherapy with and without pembrolizumab in 749 PD-L1-unselected EGC patients (73% ESCC, 16% EAC, 11% GEJC). For patients with ESCC with CPS ≥ 10 (50%), the median overall survival was 13.9 months for chemotherapy + IO, vs. 8.8 months for chemotherapy alone; the CPS < 10 group failed to show benefit (the FDA approval does not specify the CPS threshold in the first-line chemotherapy+IO setting for pembrolizumab, though the NCCN Guidelines and European Commission approvals do) [68]. The second first-line approval came less than a month later, on 16 April 2023, for nivolumab as studied in CheckMate 649 (1581 patients, PD-L1-unselected 100% AC: 70% GC, 18% GEJC, 12% EAC), with similar outcomes to KEYNOTE-590, including the familiar caveat that the subgroup analysis did not show benefit for patients with CPS < 5%, also leading to discordance between the FDA and European Commission approvals, with the latter approving the drug only if CPS meets or exceeds 5% [69]. Again, about a month later, another approval was granted, this time for the KEYNOTE-811 regimen, which combines pembrolizumab with chemotherapy and trastuzumab for HER2+ GEJC and GC, showing an objective response rate of 74.4% for the triple combination vs. 51.9% for hormonal therapy plus chemotherapy, and complete response rates of 11.3% vs. 3.1%, respectively [70].

Fifteen days after this, on 20 May 2021, the first FDA approval for adjuvant IO monotherapy was approved, based on the results of CheckMate 577. This regimen studied nivolumab given after a CROSS-style regimen with residual pathological disease after R0 resection, for both AC and SCC EGCs, agnostic of CPS. Adjuvant nivolumab reached a disease-free survival of 22.4 months vs. 11.0 months with placebo [71], representing a significant improvement on the still-persistent problem recognized early on by Czerny and others, namely, that an apparently successful resection (defined macroscopically, as would have been done classically) leads to early relapse and death in far too many cases.

After this flurry of firsts in mid-2021, the next paradigm-shifting approval came on 27 May 2022 for CheckMate 648, which was a three-arm trial comparing IO + chemotherapy, IO + IO, or chemotherapy alone in ESCC. The IO + chemotherapy and IO+IO (nivolumab with ipilimumab) arms had statistically similar outcomes: a median overall survival of 13.2 months vs. 12.8 months, vs. 10.7 months for chemotherapy alone. Though not explored in detail here due to space constraints, a timeline of approvals of China-originated IO is shown in Table 2, interspersed with the approvals noted above. The time frame of approvals is similar to those of international drugs, and several international trials are underway to determine global applicability. We also draw attention to the international zanidatamab (a HER2 bispecific antibody) + tislelizumab + chemotherapy study currently underway, with promising preliminary results [72].

Anti-HER2 vaccine therapy, hearkening back to the earliest IO experiments with less targeted vaccines in the early 20th century, is beginning to mature. The first overall survival data for HER-Vaxx (IMU-131) were published in the preliminary HERIZON study results, demonstrating overall survival of 13.9 months with HER-Vaxx with chemotherapy vs. 8.3 months for chemotherapy alone, in the second-line setting for advanced HER2+ GC and GEJC [73].

## 7. Barriers to Equitable Care

EGCs are globally common diseases that come with severe morbidity and mortality, but the improvements noted above are heartening in several aspects. It is notable that many improvements are achieved with multimodal care, and with advanced systemic therapies including immunotherapy. However, limited availability and the high costs associated with both multimodal care and advanced systemic therapy create a ripe setting for inequity. Additionally, improved outcomes, while much better than the alternative, are not without intrinsic costs—similar to other chronic conditions, patients living longer with cancer require longitudinal medical attention leading to an increased economic burden on patients, their families, and the healthcare system. Patients with fewer social and economic resources face even greater challenges [74].

An important variable when considering equity is access to services. One important factor in access is the divide between urban and suburban resource availability. Many chemotherapy infusion centers are at major academic centers and large hospital networks in suburban and urban areas. It is estimated that 15% to 19% of US Americans live outside of major metropolitan areas, placing a large proportion of the US population at risk of not receiving appropriate services and therapies [75]. The combined incidence rates for cancer are higher in urban areas and death rates are higher in rural areas [76]. Within rural settings, the healthcare system is also more physically spread out with fewer generalists and specialist providers. It is estimated that nonmetropolitan counties, those containing less than 50,000 people, are half as likely to have an oncologist as compared to metropolitan counties [76]. EC is particularly multidisciplinary, as shown by the evolving treatment paradigms outlined above, and a single patient may require evaluation by all of a panel of specialists including diagnostic and interventional radiologists, pathologists, medical oncologists, radiation oncologists, gastroenterologists, and oncologic surgeons (we recognize that a single physician may play multiple of these roles, depending on the custom in the country and specific institution). Even if care is established, with the appropriate multidisciplinary team assembled, increased travel costs to specialist visits and scheduled treatments introduce another barrier to receiving any recommended care. Altogether, these factors lead to an increased risk of lower quality care for those residing in rural areas.

Patients with poor health literacy face many barriers to equitable care for a multitude of reasons. For the past few decades, there has been an emphasis on reducing disparities in cancer screening, treatment plans, and enrollment in clinical trials [74,75,77,78,79]. Of recent note, there is a greater push to recognize limitations in health literacy in disparaged groups. Navigating the complex diagnostic and therapeutic concepts such as “lesion”, ”tumor”, or “cure” can lead to struggles in care decisions. Patients often do not want to express the limited understanding of their health for fears of rejection or embarrassment. It was one estimated that roughly 67 percent with poor health literacy do not want to share this with their spouse [80]. It is also difficult to truly determine health literacy as patient’s can conceal their lack of understanding towards their care. Health literacy can have a profound impact on cancer care and must be appropriately addressed early when establishing care with a new patient.

When considering equity of comprehensive cancer care, cost of treatment must be considered. Within the United States, there is limited data for the projected cost of esophageal cancer treatment because, in the US system, there is significant variability dependent upon state, insurance type, and overall coverage. A recent study performed a cost analysis of treating EAC and ESCC, utilizing data from 1998 to 2013 for Medicare-enrolled patients over 65, and found that total monthly costs during staging reached up to USD 8953 (USD 8385–USD 9485) per month and increased substantially during the 6-month terminal phase at USD 18,150 (USD 17,211–USD 19,089) per month. The highest initial phase cost was in Stage IV disease, at USD 9263 (USD 8758–USD 49,768) per month [81]. This study is not completely generalizable even within the US population as it was specifically looking at Medicare enrollees over the age of 65, and approximately 60% of EC is diagnosed above the age of 65 (therefore, more than 40% of patients are not represented) [82]. Even if this study only grossly approximates the general reality, many low-cost insurance plans may not cover all necessary care, and patients with no insurance may be faced with bills far outpacing the national average income. As standards of care become more costly by increasingly including IO and targeted therapies, it is likely the overall cost of care will continue to increase as well.

IO is expensive. A single cycle of nivolumab is estimated to cost USD 6676 in the USA (USD ~160,000 per year) [83]. If IO is one of the key steps in improving outcomes across the board, which strategies can help to improve access worldwide? While there is no one-size-fits-all strategy, there are pockets of inspiration. Many pharmaceutical companies have grant programs to increase access in the United States and other countries, and clinical trials are occasionally helpful as well (though participation in these is also often limited by insurance). These strategies do little to help those in more rural areas, where the ability to enroll in a grant program or clinical trial may be severely limited or completely unavailable. In situations where free or significantly cost-reduced drugs from the manufacturers are not available, and grant programs are more difficult to come by, creative techniques are being employed. For example, Tata Medical Center in Mumbai, India, has pioneered a strategy for the treatment of head and neck cancers that is based on the careful examination of the pharmacodynamics of IO. They developed a randomized trial that built on prior success at their own institution with a triple metronomic therapy (a combination of oral methotrexate, celecoxib, and erlotinib): with basic science and retrospective studies suggesting the possible efficacy of ultra-low-dose IO, they combined these into a regimen that used the triple metronomic therapy plus nivolumab at a 20 mg flat dose every three weeks [84]. This dosing allowed them to share vials between patients to give many more doses than would be possible with the more common dosing strategy (for comparison, CheckMate 648 gave a 240 mg flat dose every 2 weeks—over 6 weeks, this would amount to 720 mg vs. 60 mg over the same period in the Tata Medical Center strategy). The estimated costs of this study were <10% compared with typical nivolumab use in the US. The arm that received low-dose nivolumab in addition to triple metronomic therapy achieved a 1-year overall survival of 43.4%, compared to 16.3% in the arm that received triple metronomic therapy alone [84]. Perhaps a similar strategy, combining low-dose IO with whichever standard of care chemotherapy is appropriate for the pathologic subtype and geographic location, would be efficacious in EC as well, and hold the potential to greatly expand access. Less frequent dosing and better tolerated backbones may particularly benefit patients in rural areas, in which the current realistic alternative to intensive treatment may be no treatment at all. Even a less aggressive dose reduction method, such as dose down-rounding, which rounds down to the smaller vial size provided that the calculated dose is within a given percentage (e.g., 5–10%) of the goal, has been shown to have large potential cost-savings [85]. In addition to the creative use of existing drugs, autocthonic IO, such as those developed and under development in China and other countries, of which there are substantially more than a handful with FDA approval (see Table 2), may also help narrow worldwide disparity gaps. However, it seems less likely that global efforts to manufacture less expensive IO will make much difference specifically within the USA, at least in the near future. Recent efforts to obtain FDA approval for one of these drugs, China-produced sintilimab, which had a planned cost of ~60% as compared to existing IO, failed [86], though, as mentioned above, other internationalization studies with Chinese IO are underway. We remain hopeful that the continued development of competition in the IO and targeted therapeutics industry will drive costs down while demand increases, in addition to patient programs, creative yet efficacious dosing strategies, and work by legislative bodies to ensure equitable care.

## 8. Conclusions and Future Directions

The diagnosis and treatment of EC has advanced greatly over the 5000 years since its first written description, with major improvements in the last 100 years in operative technique, minimally invasive strategies, radiotherapy, chemotherapy, IO, and, most importantly, multimodal treatments. These advancements, in addition to developments in patient selection and supportive care, have led to increases in both cure rate and survival. However, there is much work to be done, including continuing to pursue clarity on optimal multimodality regimens, conscientiously incorporating IO, and expanding these advances both worldwide and across socioeconomic strata.

## Figures and Tables

**Table 1:** Early history of esophageal cancer.

Key Events in the Early History of Esophageal Cancer		
Date	Event	Notes
3000 BCE	First description of esophageal surgery, written in Egypt.	Smith Surgical Papyrus.
0 BCE	First description of EC, written in China.	Epidemiologic links to EC described for alcohol, hot drinks, and advanced age.
131–200	First descriptions of EC written in the West.	Dates are the life of Galen, Roman Greek physician who wrote extensively. Poor prognosis described.
1090–1162	First palliative methods for EC described, including esophagogastric feeding tubes.	Dates are the life of Ibn Zuhr, Arabian physician who described these methods.
1543	First detailed illustrations and descriptions of the upper gastrointestinal tract.	Vesalius, *De Humanis Corporis Fabrica.*
1690	First personal description of living with EC.	Diary of John Casaubon, English surgeon.
1770	First written Western hypothesis of the epidemiologic link between alcohol and EC.	Ernst Gottfried Gyser, “Medical inaugural dissertation on the fatal hunger, caused by callous narrowing of the esophagus, with phenomena worthy of attention which are detected in certain abdominal viscera.”
1857	First described EC operation.	Albrecht Theodor von Middeldorpf, German surgeon.
1868	Esophagoscope invented.	Adolf Kussmaul, German surgeon.
1872	First known esophagectomy.	Theodor Billroth, German surgeon, with Vincenz Czerny assisting.
1877	First known cervical esophagectomy.	Vincenz Czerny. Postoperative survival of 15 months.
1913	First known curative EC resection.	Franz Torek, United States surgeon. Postoperative survival of 12 years.
1933	First report on a series of EC resections.	Tohru Oshawa, Japanese surgeon. Eighteen resections, 56% mortality.
1947	First large report on a series of EC resections in the West.	Richard Sweet, United States surgeon. In total, 213 resections, 17% mortality, 8% 5-year survival.
1959	First report with <10% operative mortality.	Komei Nakayama, Japanese surgeon. In total, 953 resections, 5.8% mortality.
1981	First report with <5% operative mortality.	Hiroshi Akiyama, Japanese surgeon. In total, 210 resections, 1.4% mortality, 34.6% 5-year survival.

Acronyms: BCE—before common era; EC—esophageal cancer.

**Table 2:** Key clinical trials and approvals.

Key Clinical Trials and Approvals		
1981	First neoadjuvant RT trial for EC.	Launois et al. 40 Gy. Results were negative [42].
1981	First neoadjuvant CRT trial for EC.	Steiger et al. 30 Gy, 5-FU + [mitomycin-C vs. cisplatin]. pCR 31%, CRT mortality 10%, operative mortality 10% (survival reported subsequently, no benefit-Leichman et al. 1984 [44]).
1988	First perioperative chemotherapy trial for EC.	Roth et al. No benefit for cohort overall. mOS of responders 20 mo, nonresponders 6.2 mo, surgery alone 8 mo [45].
2002	First whole-cohort positive perioperative chemotherapy trial.	Lancet, United Kingdom. Cisplatin + fluorouracil. mOS 16.8 mo vs. 13.3 mo for surgery alone [53].
2010	Trastuzumab shown to have benefit for HER2+ GC and GEJC.	ToGA trial. Trastuzumab+chemotherapy. mOS 13.8 mo vs. 11.1 mo for chemotherapy alone.
2012	CROSS trial first report.	Van Hagen et al. 40 Gy, carboplatin+paclitaxel. mOS 49.4 mo vs. 24.0 mo for surgery alone [61].
22 September 2017	First FDA approval for IO for EGC.	KEYNOTE-059 [65], pembrolizumab monotherapy, approved for 3rd line. Approval was later withdrawn as pembrolizumab moved to earlier lines.
30 July 2019	First FDA approval for 2nd line IO for EGC.	KEYNOTE-181, pembrolizumab monotherapy. ESCC with CPS ≥ 10. mOS 8.2 mo vs. 7.1 mo for chemotherapy [66].
10 June 2020	First FDA approval for 2nd line IO for EGC, agnostic of CPS.	ATTRACTION-3, nivolumab monotherapy. ESCC. mOS 10.9 mo vs. 8.4 mo for chemotherapy [67].
19 June 2020	First NMPA approval for locally-produced IO, 2nd line camrelizumab for ESCC.	ESCORT, camrelizumab monotherapy. ESCC. mOS 8.3 mo vs. 6.2 mo for chemotherapy.
15 January 2021	First FDA approval for antibody drug conjugate in EGC.	DESTINY-Gastric01, fam-trastuzumab deruxtecan-nxki. EGC, AC, HER2+, 2nd line. mOS 12.5 mo vs. 8.4 mo for chemotherapy.
22 March 2021	First FDA approval for 1st line IO for EGC.	KEYNOTE-590, pembrolizumab with chemotherapy, EGC, AC and SCC, CPS agnostic. mOS 13.9 mo (ESCC w CPS ≥ 10) vs. 8.8 mo for chemotherapy alone [68].
16 April 2021	Second FDA approval for 1st line IO for EGC.	CheckMate 649, nivolumab with chemotherapy, AC only, CPS agnostic. mOS 13.8 vs. 11.1 mo for chemotherapy alone [69].
05 May 2021	First FDA approval for 1st line IO + chemotherapy + HER2-targeted therapy.	KEYNOTE-811, pembrolizumab + trastuzumab + chemotherapy. ORR 74.4% vs. 51.9% for trastuzumab + chemotherapy alone. CR 11.3% vs. 3.1%, respectively [70].
20 May 2021	First FDA approval for adjuvant IO monotherapy.	CheckMate 577, nivolumab after CROSS, EGC, AC and SCC, CPS agnostic. ESCC mDFS 29.7 mo vs. 11 mo for placebo, EAC 19.4 mo vs. 11 mo [71].
10 December 2021	NMPA approval for 1st line camrelizumab + chemotherapy for ESCC.	ESCORT-1st, camrelizumab with chemotherapy. mOS 15.3 mo vs. 12.0 mo for chemotherapy alone.
21 February 2022	NMPA approval for 1st line tislelizumab monotherapy for GC and GEJC.	Based on phase I/II studies.
13 April 2022	NMPA approval for 2nd line tislelizumab monotherapy for ESCC.	RATIONALE-302, tislelizumab monotherapy vs. chemotherapy, ESCC, PD-L1 agnostic. mOS 8.6 mo vs. 6.3 mo for chemotherapy.
19 May 2022	NMPA approval for 1st line tislelizumab + chemotherapy for ESCC.	RATIONALE-306, tislelizumab with chemotherapy, ESCC, PD-L1 agnostic. mOS 17.2 mo vs. 10.6 mo for chemotherapy alone.
27 May 2022	First FDA approval for 1st line dual IO.	CheckMate 648, nivolumab with ipilimumab, ESCC, PD-L1 ≥ 1%. mOS 13.2 mo for IO + chemotherapy vs. 12.8 mo for IO + IO vs. 10.7 mo for chemotherapy alone.
20 June 2022	NMPA approval for 1st line sintilimab + chemotherapy for GC and GEJC, agnostic of CPS.	ORIENT-16, sintilimab + chemotherapy vs. chemotherapy, AC. For CPS ≥ 5, mOS 19.2 mo vs. 12.9 mo for chemotherapy alone. For unselected CPS, mOS 15.2 mo vs. 12.3 mo for chemotherapy alone.
24 January 2023	First OS data available for HER2 vaccine therapy.	HERIZON study, HER-Vaxx (IMU-131) + chemotherapy, metastatic or advanced HER2+ GC and GEJC. mOS 13.9 mo for vaccine+chemotherapy vs. 8.3 mo for chemotherapy alone [73].
24 February 2023	NMPA approval for 1st line tislelizumab + chemotherapy for GC and GEJC.	RATIONALE-305, tislelizumab + chemotherapy vs. chemotherapy, AC. For PD-L1 ≥5%, mOS 17.2 mo for IO + chemotherapy vs. 12.6 mo for chemotherapy alone.

Acronyms and abbreviations: CPS—combined positive score; (C)RT—(chemo)radiotherapy; (E)AC—(esophageal) adenocarcinoma; EC—esophageal cancer; EGC—esophagogastric cancers; (E)SCC—(esophageal) squamous cell carcinoma; FDA—United States Food and Drug Administration; IO—immuno-oncologic therapy; pCR—pathologic complete response; PD-L1—programmed death-ligand 1; mDFS—median disease-free survival; mo—month(s); mOS—median overall survival; NMPA—China’s National Medical Products Administration; ORR—overall response rate.

## Data Availability

The data used for the findings of the current study are available on request from the corresponding author.

## Abbreviations

BE	Barrett’s Esophagus
EC	Esophageal Cancer
EAC	Esophageal Adenocarcinoma
ESCC	Esophageal Squamous Cell Cancer
GC	Gastric cancer
GEJC	Gastroesophageal junction cancer
IO	Immuno-oncologic (immunotherapy)
PD-1/PD-L1/2	Programmed-cell death 1/Programmed-cell death-ligand 1/2
CTLA-4	Cytotoxic T-lymphocyte-associated antigen 4
TME	Tumor Micro-Environment
CPS	Combined Positive Score
MSI	Microsatellite Instability
MSS	Microsatellite Stable
PFS	Progression-free survival
DFS	Disease-free survival
OS	Overall Survival
dMMR	Deficient MisMatch Repair
pCR	Pathologic complete response
